# The Protective Effect of Exogenous Ascorbic Acid on Photosystem Inhibition of Tomato Seedlings Induced by Salt Stress

**DOI:** 10.3390/plants12061379

**Published:** 2023-03-20

**Authors:** Xianjun Chen, Hongwei Han, Yundan Cong, Xuezhen Li, Wenbo Zhang, Wenliang Wan, Jinxia Cui, Wei Xu, Ming Diao, Huiying Liu

**Affiliations:** 1 Department of Horticulture, Agricultural College, Shihezi University, Shihezi 832003, China; 2 Key Laboratory of Special Fruits and Vegetables Cultivation Physiology and Germplasm Resources Utilization of Xinjiang Production and Contruction Crops, Shihezi 832003, China; 3 Key Laboratory of Horticulture Crop Genomics and Genetic Improvement in Xinjiang, Institute of Horticultural Crops, Xinjiang Academy of Agricultural Sciences, Urumqi 830000, China

**Keywords:** ascorbic acid, tomato, salt stress, photosystem inhibition, photoprotection

## Abstract

This study investigated the protective effects of exogenous ascorbic acid (AsA, 0.5 mmol·L^−1^) treatment on salt-induced photosystem inhibition in tomato seedlings under salt stress (NaCl, 100 mmol·L^−1^) conditions with and without the AsA inhibitor lycorine. Salt stress reduced the activities of photosystem II (PSII) and PSI. AsA treatment mitigated inhibition of the maximal photochemical efficiency of PSII (*F*_v_/*F*_m_), maximal P700 changes (*P*_m_), the effective quantum yields of PSII and I [Y(II) and Y(I)], and non-photochemical quenching coefficient (*NPQ*) values under salt stress conditions both with and without lycorine. Moreover, AsA restored the balance of excitation energy between two photosystems (*β/α*-1) after disruption by salt stress, with or without lycorine. Treatment of the leaves of salt-stressed plants with AsA with or without lycorine increased the proportion of electron flux for photosynthetic carbon reduction [*J*e(PCR)] while decreasing the O_2_-dependent alternative electron flux [*J*a(O_2_-dependent)]. AsA with or without lycorine further resulted in increases in the quantum yield of cyclic electron flow (CEF) around PSI [Y(CEF)] while increasing the expression of antioxidant and AsA–GSH cycle-related genes and elevating the ratio of reduced glutathione/oxidized glutathione (GSH/GSSG). Similarly, AsA treatment significantly decreased the levels of reactive oxygen species [superoxide anion (O_2_^−^) and hydrogen peroxide (H_2_O_2_)] in these plants. Together, these data indicate that AsA can alleviate salt-stress-induced inhibition of PSII and PSI in tomato seedlings by restoring the excitation energy balance between the photosystems, regulating the dissipation of excess light energy by CEF and *NPQ*, increasing photosynthetic electron flux, and enhancing the scavenging of reactive oxygen species, thereby enabling plants to better tolerate salt stress.

## 1. Introduction

Soil salinization and secondary salinization threaten sustainable agriculture and ecological integrity throughout the world [1,2]. Salinization is also aggravated by climate change, over-fertilization, and over-irrigation. Tomatoes (*Solanum lycopersicum* L) are widely grown in open-field and facility production settings. While tomato plants do show some degree of salt tolerance, they are nevertheless susceptible to salt stress, which inhibits both photosynthesis and production [3,4]. Thus, new approaches are needed to alleviate the effects of salt stress on tomatoes to ensure the efficient production of tomato crops.

Photosynthesis is driven by light energy, but stress conditions can reduce the conversion of absorbed light energy and associated CO_2_ assimilation, resulting in the generation of excess light energy. If not dissipated, this excess energy can trigger the accumulation of reactive oxygen species (ROS), leading to photo-oxidative damage to the photosystem and contributing to increasingly severe photoinhibition. Plants have evolved a series of photoprotective mechanisms that can help mitigate such damage. For example, plants can reduce absorption of light energy by altering the leaf angle or leaf area, decreasing the antenna pigment content, and altering the numbers of reaction centers [5]. Non-photochemical quenching (NPQ) can also facilitate thermal dissipation, while cyclic electron flow (CEF) around photosystem I (PSI)can maintain an appropriate proton gradient (ΔpH) across the thylakoid membrane necessary to facilitate the dissipation of excessive excitation energy [6]. Plants can also reduce photoinhibition through photorespiration, enhanced Mehler’s reaction, state transition of the photosystem, and ROS scavenging mechanisms to regulate the distribution of excitation energy in the photosystem [7,8]. As an alternative electron pathway, CEF around PSI can regulate electron transfer rates, maintain the balance between ATP and NAPDH, and protect against excessive PSI receptor reduction, thereby decreasing rates of hydroxyl radical (·OH) formation to prevent photodamage to PSI [9,10]. This mechanism can also aid in the repair of photodamage to photosystem II (PSII) [11].

Plants can engage a range of exogenous substances to augment salt tolerance in response to increased salinity. The application of exogenous substances thus represents a promising means of improving plant salt tolerance. Ascorbic acid (AsA), also known as vitamin C, is a small-molecule antioxidant that is present at high levels in the chloroplast stroma and in other parts of plant tissues, wherein it functions as a key electron donor in redox reactions and can regulate photosynthesis by preserving photosystem integrity [12,13]. The synthesis of AsA is mediated by the enzyme L-Galactono-1, 4-lactone dehydrogenase (L-GalLDH), and the overexpression of this gene in rice leads to significant increases in endogenous AsA production and levels of the ribulose-1, 5-bisphosphate (RuBP) and carboxylase/oxygenase (Rubisco) proteins [14], while silencing of L-GalLDH reduced endogenous AsA production by 30–50%. The resultant AsA deficiency rendered rice plants more susceptible to H_2_O_2_ production and lipid peroxidation in addition to reducing total antioxidant capacity and the overall photosynthetic capacity of plants [15]. In addition, exogenous AsA was found to play a vital role in abiotic stress responses, increasing the antioxidant properties of sweet pepper and thus improving the drought resistance of the plant [16]. Moreover, exogenous AsA can increase endogenous AsA, proline, and photosynthetic pigment levels, thus improving heat tolerance in tomato seedlings [17]. AsA can also reduce cold-associated oxidative damage by reducing lipid peroxidation, electrolyte leakage, and hydrogen peroxide (H_2_O_2_) production [18]. The benefits of AsA have also been detected under stress conditions induced by heavy metals [19], high nitrate levels [20], and high salt levels [21], showing that AsA can directly influence the antioxidant capacity and photosynthetic activity of plants.

Our previous study using fast OJIP fluorescence kinetics and JIP-test analyses showed that exogenous AsA treatment increased endogenous AsA levels and alleviated PSII photoinhibition, thus promoting tomato seedling growth under salt stress conditions [22]. However, these findings reflected PSII photochemical changes before the start of the dark reaction, and little is known of the utilization of light energy by the photosystems after carbon assimilation is initiated. PSII is generally regarded as being more sensitive than PSI, and it is thus considered the primary site of photoinhibition. Despite this, some reports have found that low-temperature and low-light conditions damage PSI more severely than PSII. Indeed, Terashima et al. [23] reported that in cucumber leaves exposed to low temperatures, PSI, rather than PSII, was the primary site of photoinhibition. Little research has been conducted on how PSI functions in tomato seedlings under salt stress [24], and the role of AsA as a regulator of photoinhibition in this setting is not well understood. Thus, the present study investigated the effects of exogenous AsA application and an inhibitor of AsA synthesis, lycorine, on tomato seedlings, examining the activity, light energy partitioning, and electron transport in PSI and PSII under conditions of salt stress. PSII electron flow partitioning, CEF around PSI (CEF-PSI), and ROS scavenging activity were also investigated.

## 2. Results

### 2.1. PSI and PSII Activity Levels

**Figure 1:**
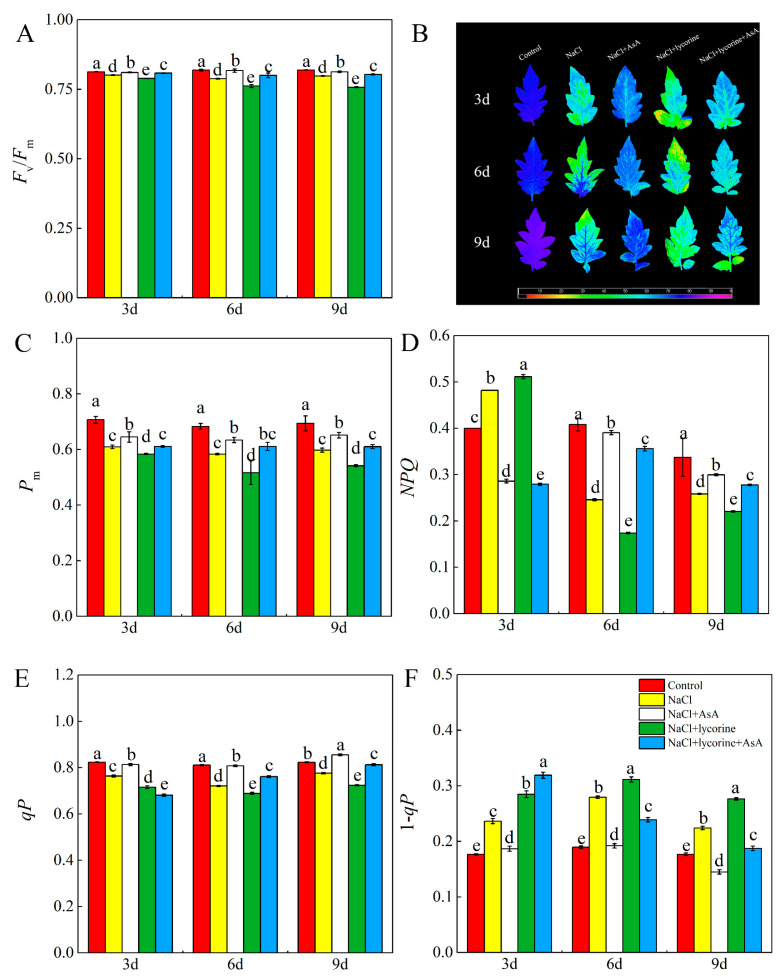
Values of the maximal photochemical efficiency of PSII (*F*_v_/*F*_m_) (**A**), false-color images of *F*_v_/*F*_m_ (**B**), maximal P700 changes (*P*_m_) (**C**), non-photochemical quenching coefficient (*NPQ*) (**D**), photochemical quenching coefficient (*qP*) (**E**), and PSII excitation pressure (1–*qP*) (**F**) in leaves of salt-stressed tomato seedlings with or without exogenous reduced ascorbic acid (AsA) and lycorine (AsA synthesis inhibitor) spraying. Abbreviations 3d, 6d, and 9d represent the third, sixth, and ninth day after treatment, respectively. Control, no added NaCl and sprayed with distilled water; NaCl, added 100 mmol·L^–1^ NaCl and sprayed with distilled water; NaCl + AsA, added 100 mmol·L^–1^ NaCl and sprayed with 0.5 mmol·L^–1^ AsA; NaCl + lycorine, added 100 mmol·L^–1^ NaCl and sprayed with 0.25 mmol·L^–1^ lycorine; NaCl + lycorine + AsA, added 100 mmol·L^–1^ NaCl and sprayed with 0.25 mmol·L^–1^ lycorine plus 0.5 mmol·L^–1^ AsA. Values are means ± SD (*n* = 3). Values with a different letter within a sampling date are significantly different (*p* < 0.05).

**Figure 2:**
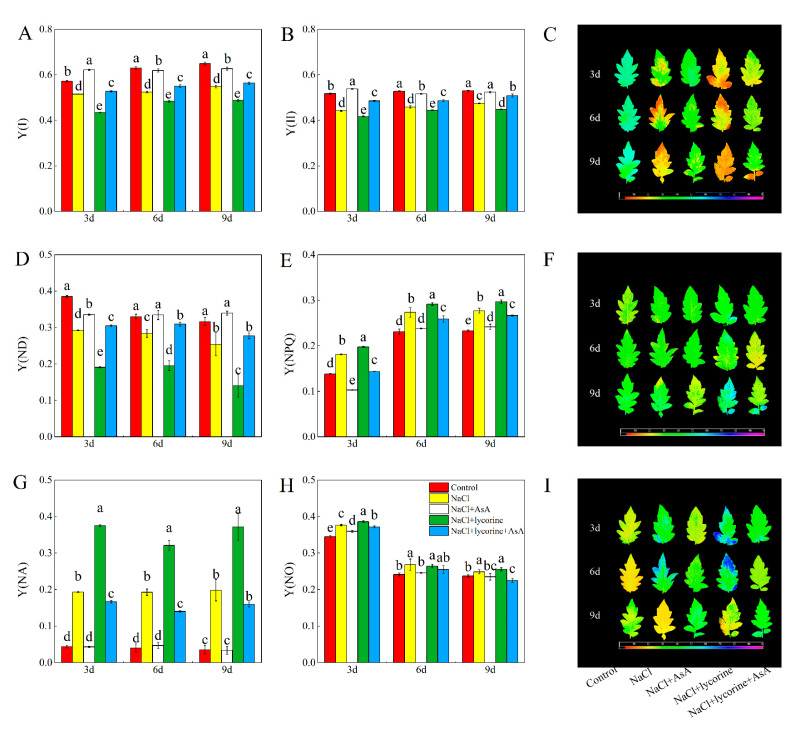
Values of the effective quantum yield of PSI (Y(I)) (**A**), effective quantum yield of PSII (Y(II)) (**B**), false-color images of Y(II) (**C**), fraction of over P700 that is oxidized in a given state (Y(ND)) (**D**), the quantum yield of regulated non-photochemical energy dissipation of PSII (Y(NPQ)) (**E**), false-color images of Y (NPQ) (**F**), fraction of over P700 that cannot be oxidized in a given state (Y(NA)) (**G**), the quantum yield of non-regulated energy dissipation of PSII (Y(NO)) (**H**), and false-color images of Y(NO) (**I**) in leaves of salt-stressed tomato seedlings with or without exogenous reduced ascorbic acid (AsA) and lycorine (AsA synthesis inhibitor) spraying. Abbreviations 3d, 6d, and 9d represent the third, sixth, and ninth day after treatment, respectively. Control, no added NaCl and sprayed with distilled water; NaCl, added 100 mmol·L^–1^ NaCl and sprayed with distilled water; NaCl + AsA, added 100 mmol·L^–1^ NaCl and sprayed with 0.5 mmol·L^–1^ AsA; NaCl + lycorine, added 100 mmol·L^–1^ NaCl and sprayed with 0.25 mmol·L^–1^ lycorine; NaCl + lycorine + AsA, added 100 mmol·L^–1^ NaCl and sprayed with 0.25 mmol·L^–1^ lycorine plus 0.5 mmol·L^–1^ AsA. Values are means ± SD (*n* = 3). Values with a different letter within a sampling date are significantly different (*p* < 0.05).

As shown in Figure 1A, NaCl treatment led to significant reductions in *F*_v_/*F*_m_ ratios throughout the study period. In Figure 1B, *F*_v_/*F*_m_ also were shown in false-color code-based images. Under NaCl treatment, leaf colors shifted from blue to green, representing a reduction in the *F*_v_/*F*_m_ ratios. The false-color images of *F*_v_/*F*_m_ showed a trend that was consistent with the *F*_v_/*F*_m_ ratios, indicating inhibition of PSII. The Y(II) and *qP* values also declined throughout the study period relative to the controls, while the 1–*qP*, Y(NPQ), and Y(NO) values showed the opposite trend. The false-color images of Y(II), Y(NPQ), and Y(NO) (Figure 2C,F,I) were consistent with their value (Figure 2B,E,H). On day three, the *NPQ* of the NaCl-treated plants was increased relative to control plants but decreased on days six and nine post-treatment. Seedlings in the NaCl + AsA treatment group showed significant increases in leaf *F*_v_/*F*_m_, Y(II), and *qP* values of 3.7–19.2%, 10.4–21.6%, and 6.5–12.1%, respectively, compared with the NaCl-treatment group, together with significant decreases in *NPQ* (on day three), 1–*qP*, Y(NPQ), and Y(NO). Significant decreases in *F*_v_/*F*_m_, *qP*, *NPQ,* and Y(II) in NaCl + lycorine-treated plants relative to those treated with only NaCl were observed on days six and nine, while NaCl + lycorine + AsA treatment reversed the effects of NaCl + lycorine on these indices. NaCl treatment was also associated with significant increases in Y(NA) and significant reductions in *P*_m_, Y(I), and Y(ND) relative to control seedlings, indicating PSI inhibition (Figure 1 and Figure 2). NaCl + lycorine treatment significantly decreased Y(I) and Y(ND) by 7.6–15.8% and 31.1–44.6%, respectively, relative to NaCl treatment, whereas Y(NA) remained elevated relative to NaCl treatment at all time points. However, AsA treatment was sufficient to reverse the decreases in Y(I) and Y(ND) values, while AsA further reduced Y(NA) values in these seedlings relative to the NaCl and NaCl + lycorine treatment groups.

### 2.2. The Allocation of Absorbed Light Engery between PSI and PSII

**Figure 3:**
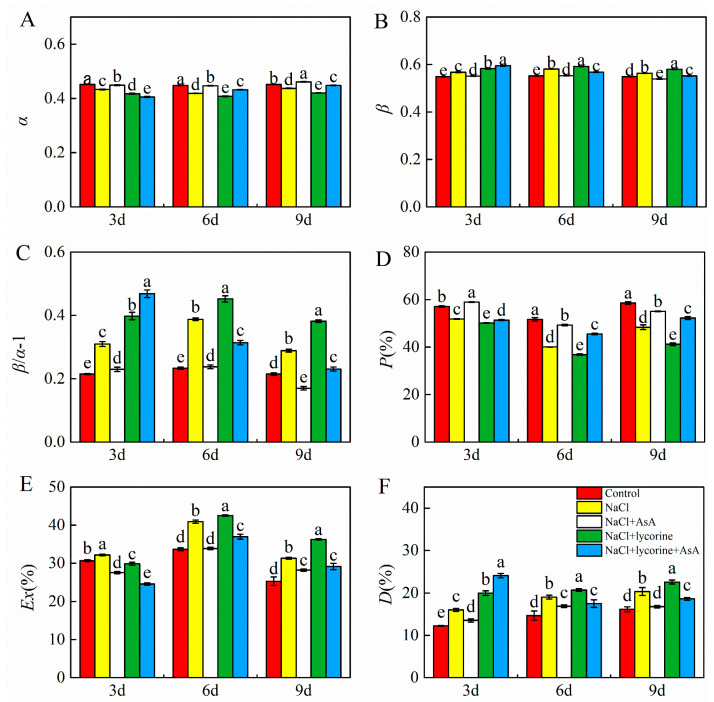
Values of the photon activity distribution coefficients of PSI (*α*) (**A**), the photon activity distribution coefficients of PSII (*β*) (**B**), the relative deviation from full balance (β/α–1) between PSI and PSII (*β/α*−1) (**C**), the fraction of photon energy absorbed in PSII antennae utilized for photosynthetic electron transport (*p*) (**D**), the estimate of the fraction of excess excitation energy that is neither dissipated in the PSII antennae nor utilized for photochemistry (*Ex*) (**E**), and the fraction of photon energy absorbed in PSII antennae and dissipated via thermal energy in the antenna (*D*) (**F**) in leaves of salt-stressed tomato seedlings with or without exogenous reduced ascorbic acid (AsA) and lycorine (AsA synthesis inhibitor) spraying. Abbreviations 3d, 6d, and 9d represent the third, sixth, and ninth day after treatment, respectively. Control, no added NaCl and sprayed with distilled water; NaCl, added 100 mmol·L^–1^ NaCl and sprayed with distilled water; NaCl + AsA, added 100 mmol·L^–1^ NaCl and sprayed with 0.5 mmol·L^–1^ AsA; NaCl + lycorine, added 100 mmol·L^–1^ NaCl and sprayed with 0.25 mmol·L^–1^ lycorine; NaCl + lycorine + AsA, added 100 mmol·L^–1^ NaCl and sprayed with 0.25 mmol·L^–1^ lycorine plus 0.5 mmol·L^–1^ AsA. Values are means ± SD (*n* = 3). Values with a different letter within a sampling date are significantly different (*p* < 0.05).

Relative to the control, NaCl stress was associated with significant reductions in *α* and *p* and significant increases in *β*, *β/α*−1, *Ex*, and *D* in tomato seedling leaves (Figure 3). The application of AsA resulted in significant improvements in *α* and *p* as well as significant reductions in *β*, *β/α*−1, *Ex,* and *D* in these NaCl-exposed plants on days three, six, and nine. Conversely, NaCl + lycorine treatment reduced the *α* and *p* values in tomato leaves, while the *β*, *β/α*-1, and *Ex* values in leaves under NaCl + lycorine + AsA treatment conditions were decreased relative to the NaCl + lycorine treatment conditions at all analytical time points.

### 2.3. PSII Electron Flux Distributions

**Figure 4:**
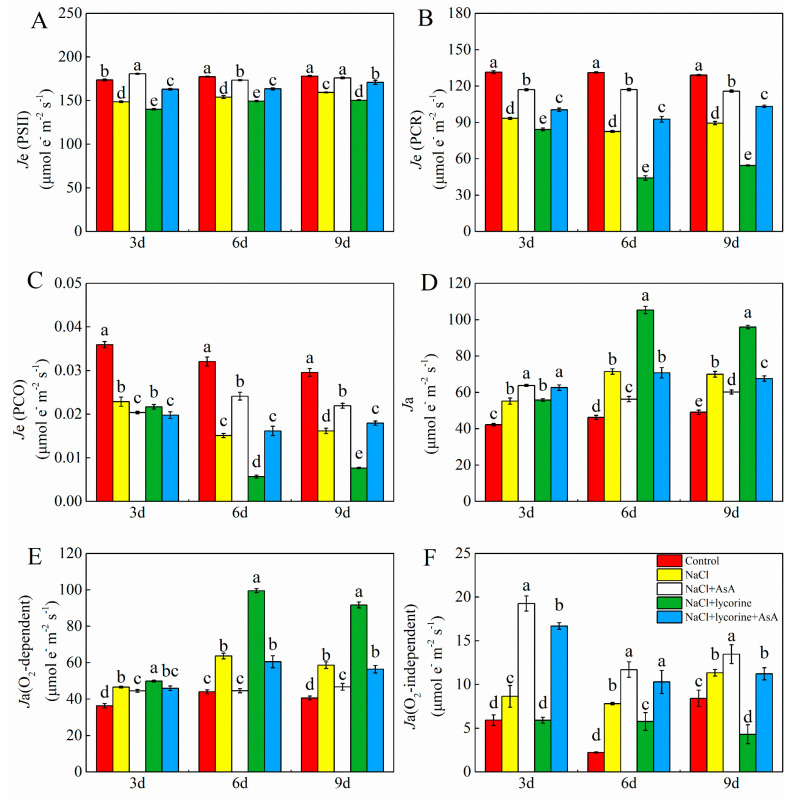
Values of the rate of electron transport through PSII *J*e(PSII) (**A**), electron flux for the photosynthetic carbon reduction cycle (*J*e(PCR)) (**B**), electron flux for photorespiration (*J*e(PCO)) (**C**), alternative electron flux (*J*a) (**D**), the O_2_-dependent alternative electron flux (*J*a(O_2_-dependent)) (**E**), and the O_2_-independent alternative electron flux (*J*a(O_2_-independent)) (**F**) in leaves of salt-stressed tomato seedlings with or without exogenous reduced ascorbic acid (AsA) and lycorine (AsA synthesis inhibitor) spraying. Abbreviations 3d, 6d, and 9d represent the third, sixth, and ninth day after treatment, respectively. Control, no added NaCl and sprayed with distilled water; NaCl, added 100 mmol·L^–1^ NaCl and sprayed with distilled water; NaCl + AsA, added 100 mmol·L^–1^ NaCl and sprayed with 0.5 mmol·L^–1^ AsA; NaCl + lycorine, added 100 mmol·L^–1^ NaCl and sprayed with 0.25 mmol·L^–1^ lycorine; NaCl + lycorine + AsA, added 100 mmol·L^–1^ NaCl and sprayed with 0.25 mmol·L^–1^ lycorine plus 0.5 mmol·L^–1^ AsA. Values are means ± SD (*n* = 3). Values with a different letter within a sampling date are significantly different (*p* < 0.05).

NaCl treatment was associated with significant reductions in *J*e (PSII), *J*e (PCR), and *J*e (PCO) in tomato leaves together with significant increases in *J*a, *J*a (O_2_-dependent), and *J*a(O_2_-independent) relative to control plants (Figure 4). NaCl + AsA treatment reversed the impact of NaCl treatment on *J*e (PSII), *J*e (PCR), and *J*a (O_2_-dependent), with these values rising by 10.4–21.6%, 25.3–41.8%, and 18.8–122.8%, respectively, relative to NaCl treatment, whereas *J*a (O_2_-dependent) decreased significantly by 4.5–30.0% throughout the study period. NaCl + lycorine treatment resulted in further decreases in *J*e (PSII), *J*e (PCR), and *J*e (PCO) as well as increases in *J*a and *J*a (O_2_-dependent). However, combined AsA treatment was sufficient to weaken the adverse impacts of NaCl + lycorine treatment on these parameters.

**Figure 5:**
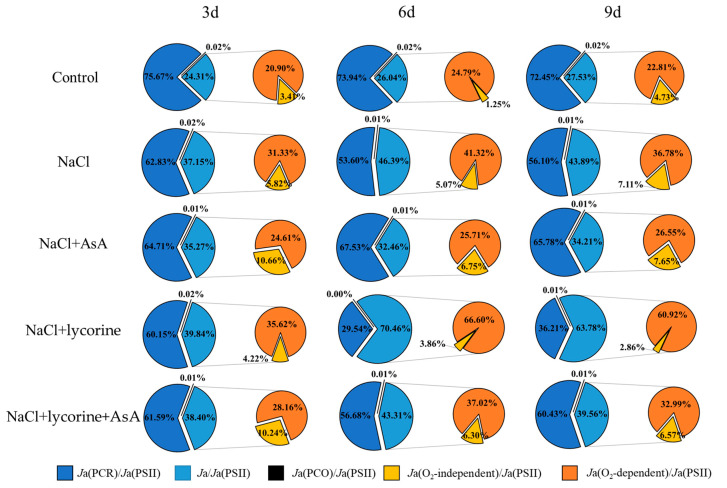
Ratio of *J*e(PCR)/*J*e(PSII), *J*e(PCO)/*J*e(PSII), *J*a/*J*e(PSII), *J*a(O_2_-dependent)/*J*e(PSII), and *J*a(O_2_-independent)/*J*e(PSII) in leaves of salt-stressed tomato seedlings with or without exogenous reduced ascorbic acid (AsA) and lycorine (AsA synthesis inhibitor) spraying. Abbreviations 3d, 6d, and 9d represent the third, sixth, and ninth day after treatment, respectively. Control, no added NaCl and sprayed with distilled water; NaCl, added 100 mmol·L^–1^ NaCl and sprayed with distilled water; NaCl + AsA, added 100 mmol·L^–1^ NaCl and sprayed with 0.5 mmol·L^–1^ AsA; NaCl + lycorine, added 100 mmol·L^–1^ NaCl and sprayed with 0.25 mmol·L^–1^ lycorine; NaCl + lycorine + AsA, added 100 mmol·L^–1^ NaCl and sprayed with 0.25 mmol·L^–1^ lycorine plus 0.5 mmol·L^–1^ AsA. Values are means ± SD (*n* = 3).

Treatment with NaCl resulted in significant reductions in the *J*e (PCR)/*J*e (PSII) and *J*e (PCO)/*J*e (PSII) ratios, while significantly increasing the *J*a/*J*e (PSII), *J*a(O_2_-independent)/*J*e (PSII), and *J*a (O_2_-dependent)/*J*e (PSII) ratios at all sampling time points relative to control seedlings (Figure 5). NaCl + AsA treatment was sufficient to reverse the impact of NaCl on the *J*e (PCR)/*J*e (PSII), *J*e (PCO)/*J*e (PSII), *J*a/*J*e (PSII), and *J*a (O_2_-dependent)/*J*e (PSII) ratios while promoting an increase in the *J*a (O_2_-independent)/*J*e (PSII) ratio. Lycorine treatment reduced the *J*e (PCR)/*J*e (PSII) and *J*a (O_2_-independent)/*J*e (PSII) ratios at all time points, decreased the *J*e (PCO)/*J*e (PSII) ratio on days six and nine, and resulted in a significant increase in *J*a/*J*e (PSII) and *J*a (O_2_-dependent)/*J*e (PSII) ratios at all time points in these NaCl-exposed seedlings. Significantly higher *J*e (PCR)/*J*e (PSII), *J*e (PCO)/*J*e (PSII), and *J*a (O_2_-independent)/*J*e (PSII) ratios were observed under NaCl + lycorine + AsA treatment conditions at all time points relative to NaCl + lycorine treatment conditions, whereas *J*a/*J*e (PSII) and *J*a (O_2_-dependent)/*J*e (PSII) were decreased.

### 2.4. CEF-PSI Analyses

**Figure 6:**
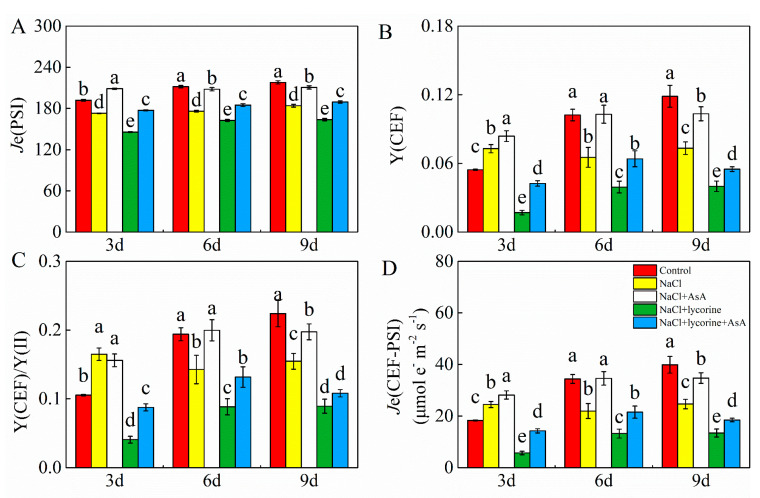
Values of the rate of electron transport through PSI (*J*e(PSI)) (**A**), the quantum yield of cyclic electron flow (CEF) around PS I (Y(CEF)) (**B**), Y(CEF)/Y (II) (**C**), and the electron flux through CEF-PSI (*J*e(CEF-PSI)) (**D**) in leaves of salt-stressed tomato seedlings with or without exogenous reduced ascorbic acid (AsA) and lycorine (AsA synthesis inhibitor) spraying. Abbreviations 3d, 6d, and 9d represent the third, sixth, and ninth day after treatment, respectively. Control, no added NaCl and sprayed with distilled water; NaCl, added 100 mmol·L^–1^ NaCl and sprayed with distilled water; NaCl + AsA, added 100 mmol·L^–1^ NaCl and sprayed with 0.5 mmol·L^–1^ AsA; NaCl + lycorine, added 100 mmol·L^–1^ NaCl and sprayed with 0.25 mmol·L^–1^ lycorine; NaCl + lycorine + AsA, added 100 mmol·L^–1^ NaCl and sprayed with 0.25 mmol·L^–1^ lycorine plus 0.5 mmol·L^–1^ AsA. Values are means ± SD (*n* = 3). Values with a different letter within a sampling date are significantly different (*p* < 0.05).

As shown in Figure 4 and Figure 6, electron transport rates for both PSI and PSII [*J*e (PSII) and *J*e (PSI)] were significantly decreased in response to NaCl and NaCl + lycorine treatment relative to control and NaCl treatment, respectively, whereas AsA promoted *J*e (PSII) and significantly increased *J*e (PSI) in the leaves of these tomato seedlings irrespective of the addition of lycorine. Salt stress was also associated with the inhibition of LEF and the stimulation of the quantum yield of CEF around PSI [Y (CEF)], the ratio of the quantum yield of CEF to Y(II) [Y (CEF)/Y (II)], and electron flux through CEF-PSI [(*J*e (CEF-PSI)] on day three post-treatment, but these indices declined significantly on days six and nine after treatment. Relative to seedlings in the NaCl or NaCl + lycorine groups, those subjected to AsA treatment exhibited significant increases in Y (CEF) by 15.1–57.7% and 37.5–150.3%, Y (CEF)/Y (II) by 27.6–39.9% and 21.1–114.9%, and *J*e (CEF-PSI) by 15.1–57.7% and 37.5–150.3%, respectively, throughout the study period.

### 2.5. ROS Metabolism and Oxidative Damage Analyses

**Figure 7:**
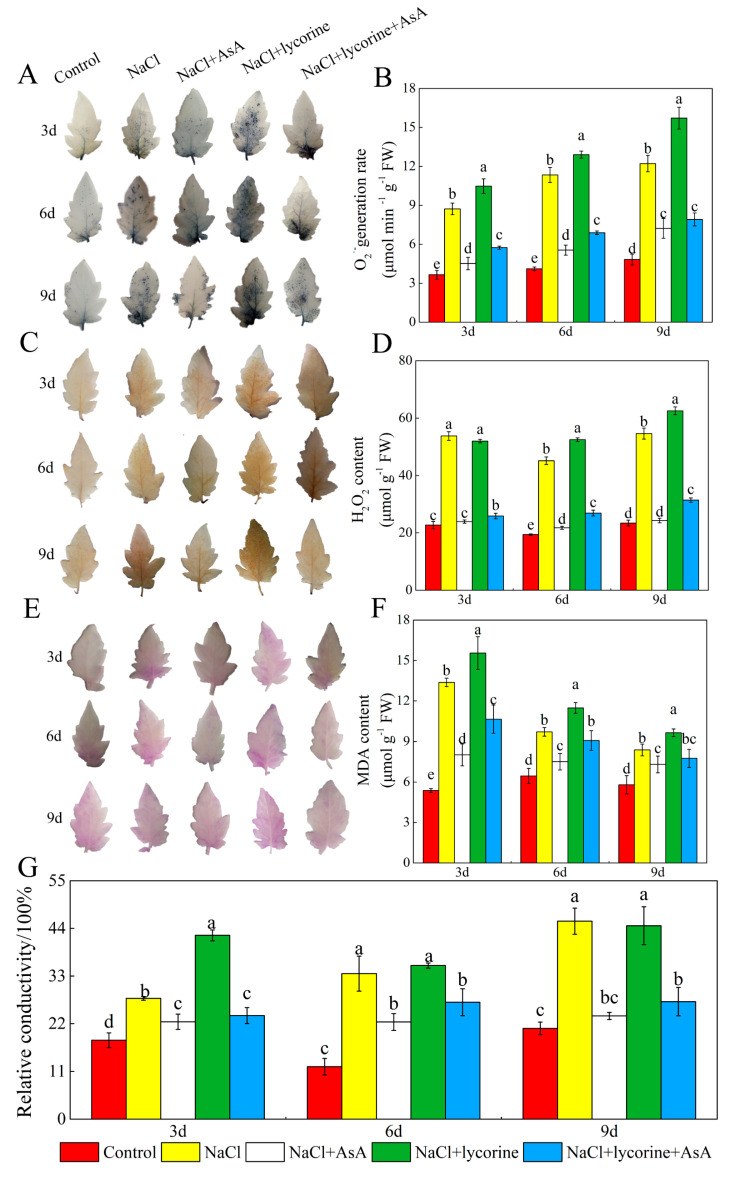
Values of histochemical detection of superoxide anion (O_2_^−^) (**A**), O^·−^ content (**B**), histochemical detection of hydrogen peroxide (H_2_O_2_) (**C**), H_2_O_2_ content (**D**), histochemical detection of MDA (**E**), MDA content (**F**), and relative conductivity (**G**) in leaves of salt-stressed tomato seedlings with or without exogenous reduced ascorbic acid (AsA) and lycorine (AsA synthesis inhibitor) spraying. Abbreviations 3d, 6d, and 9d represent the third, sixth, and ninth day after treatment, respectively. Control, no added NaCl and sprayed with distilled water; NaCl, added 100 mmol·L^–1^ NaCl and sprayed with distilled water; NaCl + AsA, added 100 mmol·L^–1^ NaCl and sprayed with 0.5 mmol·L^–1^ AsA; NaCl + lycorine, added 100 mmol·L^–1^ NaCl and sprayed with 0.25 mmol·L^–1^ lycorine; NaCl + lycorine + AsA, added 100 mmol·L^–1^ NaCl and sprayed with 0.25 mmol·L^–1^ lycorine plus 0.5 mmol·L^–1^ AsA. Values are means ± SD (*n* = 3). Values with a different letter within a sampling date are significantly different (*p* < 0.05).

Salt stress induced an increase in the O_2_^−^ generation rate, MDA and H_2_O_2_ content, and relative conductivity in the tomato leaves. Relative to NaCl-treated plants, plants treated with NaCl + AsA treatment showed O_2_^−^ generation rates that were 48.3%, 51.5%, and 40.9% lower at the three sampling time points, with significant concomitant 40.0–55.5%, 22.8–51.8%, and 12.8–55.5% reductions in the relative conductivity and levels of H_2_O_2_ and MDA in leaves of NaCl + AsA-treated seedlings (Figure 7). In addition, relative to NaCl treatment, NaCl + lycorine treatment was associated with higher levels of MDA and ROS accumulation throughout the treatment period, while combined NaCl + lycorine + AsA treatment was sufficient to reverse these adverse effects of NaCl + lycorine treatment in analyzed seedlings.

### 2.6. GSH Content and the GSH/GSSG Ratio

**Figure 8:**
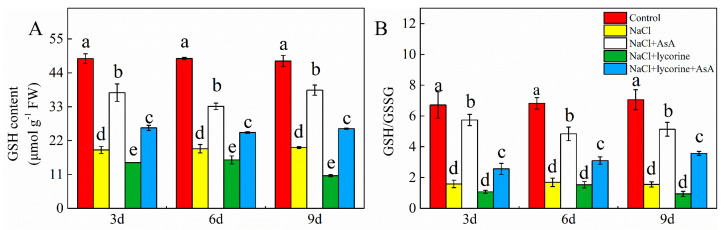
Values of reduced glutathione (GSH) content (**A**) and the ratio of GSH/GSSG (reduced glutathione/oxidized glutathione) (**B**) in leaves of salt-stressed tomato seedlings with or without exogenous reduced ascorbic acid (AsA) and lycorine (AsA synthesis inhibitor) spraying. Abbreviations 3d, 6d, and 9d represent the third, sixth, and ninth day after treatment, respectively. Control, no added NaCl and sprayed with distilled water; NaCl, added 100 mmol·L^–1^ NaCl and sprayed with distilled water; NaCl + AsA, added 100 mmol·L^–1^ NaCl and sprayed with 0.5 mmol·L^–1^ AsA; NaCl + lycorine, added 100 mmol·L^–1^ NaCl and sprayed with 0.25 mmol·L^–1^ lycorine; NaCl + lycorine + AsA, added 100 mmol·L^–1^ NaCl and sprayed with 0.25 mmol·L^–1^ lycorine plus 0.5 mmol·L^–1^ AsA. Values are means ± SD (*n* = 3). Values with a different letter within a sampling date are significantly different (*p* < 0.05).

Relative to control seedlings, salt-stressed plants showed significantly reduced GSH levels and GSH/GSSG ratio throughout the study period (Figure 8). Relative to NaCl only, NaCl + lycorine treatment was associated with significant 18.8–46.3% reductions in GSH contents without significantly impacting the GSH/GSSG ratio at any time point. Exogenous AsA administration significantly attenuated the effects of NaCl and NaCl + lycorine treatment on GSH content and the GSH/GSSG ratio at these three sampling time points.

### 2.7. Antioxidant Enzyme Gene Expression and Activity Levels

**Figure 9:**
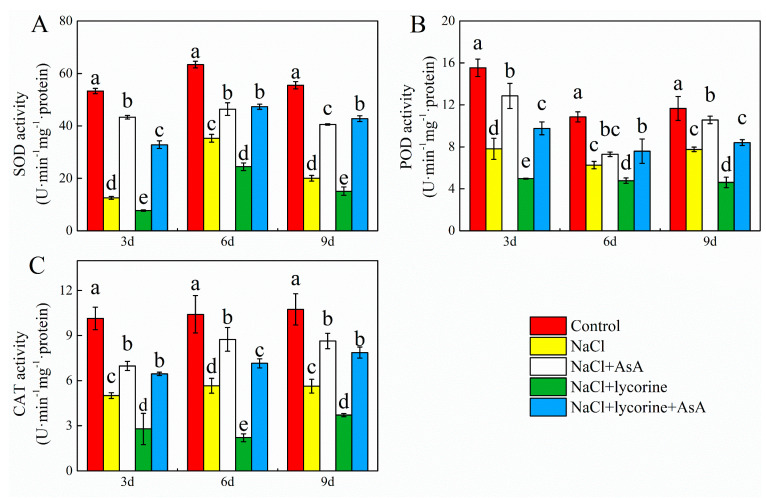
Values of superoxidase dismutase (SOD) (**A**), peroxidase (POD) (**B**), and catalase (CAT) (**C**) activity in leaves of salt-stressed tomato seedlings with or without exogenous reduced ascorbic acid (AsA) and lycorine (AsA synthesis inhibitor) spraying. Abbreviations 3d, 6d, and 9d represent the third, sixth, and ninth day after treatment, respectively. Control, no added NaCl and sprayed with distilled water; NaCl, added 100 mmol·L^–1^ NaCl and sprayed with distilled water; NaCl + AsA, added 100 mmol·L^–1^ NaCl and sprayed with 0.5 mmol·L^–1^ AsA; NaCl + lycorine, added 100 mmol·L^–1^ NaCl and sprayed with 0.25 mmol·L^–1^ lycorine; NaCl + lycorine + AsA, added 100 mmol·L^–1^ NaCl and sprayed with 0.25 mmol·L^–1^ lycorine plus 0.5 mmol·L^–1^ AsA. Values are means ± SD (*n* = 3). Values with a different letter within a sampling date are significantly different (*p* < 0.05).

**Figure 10:**
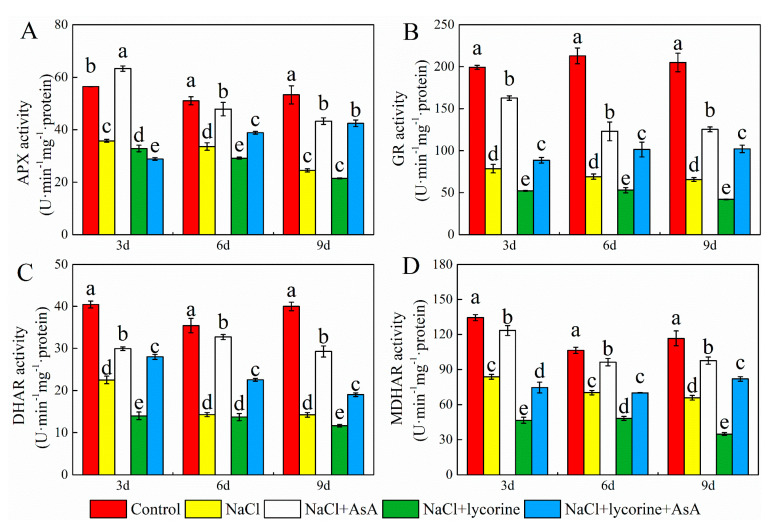
Values of ascorbate peroxidase (APX) (**A**), glutathione reductase (GR) (**B**), dehydroascorbate reductase (DHAR) (**C**), and monodehydroascorbate reductase (MDHAR) (**D**) activity in leaves of salt-stressed tomato seedlings with or without exogenous reduced ascorbic acid (AsA) and lycorine (AsA synthesis inhibitor) spraying. Abbreviations 3d, 6d, and 9d represent the third, sixth, and ninth day after treatment, respectively. Control, no added NaCl and sprayed with distilled water; NaCl, added 100 mmol·L^–1^ NaCl and sprayed with distilled water; NaCl + AsA, added 100 mmol·L^–1^ NaCl and sprayed with 0.5 mmol·L^–1^ AsA; NaCl + lycorine, added 100 mmol·L^–1^ NaCl and sprayed with 0.25 mmol·L^–1^ lycorine; NaCl + lycorine + AsA, added 100 mmol·L^–1^ NaCl and sprayed with 0.25 mmol·L^–1^ lycorine plus 0.5 mmol·L^–1^ AsA. Values are means ± SD (*n* = 3). Values with a different letter within a sampling date are significantly different (*p* < 0.05).

**Figure 11:**
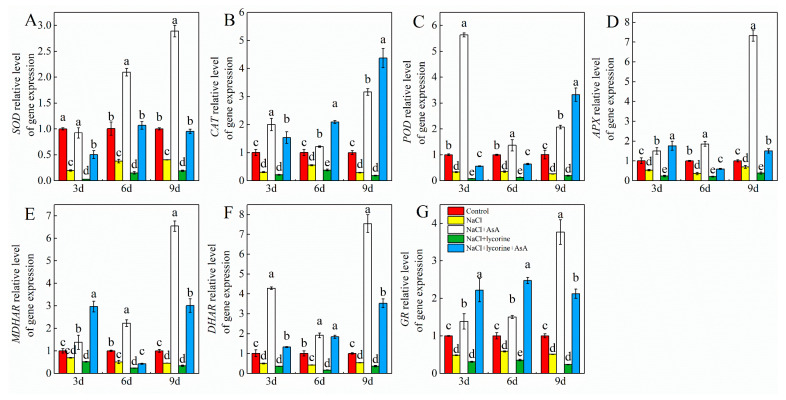
Expression of *SOD* (superoxidase dismutase gene) (**A**), *CAT* (catalase gene) (**B**), *POD* (peroxidase gene) (**C**), *APX* (ascorbate peroxidase gene) (**D**), *MDHAR* (monodehydroascorbate reductase gene) (**E**), *DHAR* (dehydroascorbate reductase gene) (**F**), and *GR* (glutathione reductase gene) (**G**) genes in leaves of salt-stressed tomato seedlings with or without exogenous reduced ascorbic acid (AsA) and lycorine (AsA synthesis inhibitor) spraying. Abbreviations 3d, 6d, and 9d represent the third, sixth, and ninth day after treatment, respectively. Control, no added NaCl and sprayed with distilled water; NaCl, addition of 100 mmol·L^–1^ NaCl and sprayed with distilled water; NaCl + AsA, added 100 mmol·L^–1^ NaCl and sprayed with 0.5 mmol·L^–1^ AsA; NaCl + lycorine, added 100 mmol·L^–1^ NaCl and sprayed with 0.25 mmol·L^–1^ lycorine; NaCl + lycorine + AsA, added 100 mmol·L^–1^ NaCl and sprayed with 0.25 mmol·L^–1^ lycorine plus 0.5 mmol·L^–1^ AsA. Values are means ± SD (*n* = 3). Values with a different letter within a sampling date are significantly different (*p* < 0.05).

Relative to control seedlings, NaCl treatment was associated with significant reductions in SOD, POD, CAT, APX, GR, DHAR, and MDHAR activity on days three, six, and nine (Figure 9 and Figure 10). Consistently, significant decreases in the expression of the genes encoding all these enzymes were observed at all three sampling time points in salt-stress-exposed seedlings relative to control seedlings (Figure 11). The expression and activity of these enzymes were significantly enhanced and decreased, respectively, upon NaCl + AsA and NaCl + lycorine treatment at all time points relative to NaCl treatment alone. However, combined NaCl + AsA + lycorine treatment reversed the deleterious effects of NaCl + lycorine treatment on such enzymatic activity and gene expression levels in these seedlings.

## 3. Materials and Methods

### 3.1. Plant Materials and Treatment Conditions

Tomato seeds (Ligeer 87-5) were incubated at 28 °C on moist filter paper for 2 days in an incubator, after which they were sown in a plastic dish containing peat and vermiculite (1:1). At the two-true-leaf stage, seedlings of uniform size were transplanted into 12-L black plastic containers (*n* = 6/container). The containers were filled with 10 L of Hoagland nutrient solution with oxygen. After 7 days, the plants were treated by adding NaCl to the nutrient solution and/or spraying AsA and/or the AsA synthase inhibitor (lycorine) on the leaves of the plants in the following combinations: (1) untreated control plants; (2) 100 mmol·L^−1^ NaCl (NaCl group); (3) 100 mmol·L^−1^ NaCl + 0.5 mmol·L^−1^ AsA (NaCl + AsA group); (4) 100 mmol·L^−1^ NaCl + 0.25 mmol·L^−1^ lycorine (NaCl + lycorine group); and (5) 100 mmol·L^−1^ NaCl + 0.25 mmol·L^−1^ lycorine + 0.5 mmol·L^−1^ AsA (NaCl + lycorine +AsA group). The AsA and lycorine volumes, concentrations, and application methods used herein were based on the results of our prior study [22]. Experiments were performed using randomized group assignments, with three replicates of five plants per treatment. Nutrient solutions were changed every third day and were oxygenated throughout the day. Samples were collected for analysis on days three, six, and nine of treatment.

### 3.2. Chlorophyll Fluorescence Parameters and P700 Redox State

The Maxi Imaging-Pam (Imaging-Pam, WALZ, Germany) modulated fluorescence imaging system was used to measure chlorophyll fluorescence parameters together with the Imaging Win program. PSII chlorophyll fluorescence and P700 redox states were simultaneously measured with a saturated pulse Dual-PAM-100 fluorometer and the Dual-PAM software. Following dark adaptation for 30 min, leaves were illuminated with a high-saturation light pulse (0.05 Hz) for 260 s. The instrument then automatically reported the following parameters: *F*_v_/*F*_m_, 1*–qP*, *qP*, *NPQ*, Y(NPQ), Y(II), *P*_m_, Y(I), Y(NO), Y(NA), and Y(ND) (Table 1). False-color images of *F*_v_/*F*_m_, Y(II), Y(NPQ), and Y(NO) images [from 0.000 (black) to 1.000 (purple)] were recorded and compared with Imaging Win [25].

### 3.3. Absorbed Light Energy Allocation Analyses

Absorbed light energy allocation and the distribution coefficients for excitation energy of PSI and PSII, including *D*, *p*, *E_x_*, *β*, *α*, and (*β/α* − 1), were analyzed using formulae reported previously by Demmig-Adams et al. [26] (Table 1).

### 3.4. LEF and CEF Electron Flux Transport Rate Calculations

Electron transport rates through PSII [*J*e(PSII)] and PSI [*J*e(PSI)] were computed using formulae reported previously by Miyake et al. [27]. Rubisco oxygenation rates (*V*_O_) and Rubisco carboxylation rates (*Vc*) can be analyzed using formulae reported previously by Sharkey et al. [28]. The distribution of electron fluxes through PSII, including *J*e(PCR), *J*e(PCO), *J*a, *J*a(O_2_-dependent), and *J*a(O_2_-independent), were analyzed using formulae reported previously by Krall et al. [29] (Table 2). The parameters *J*e(CEF-PSI), Y(CEF), and Y(CEF)/Y(II) were used for estimation of the extent of CEF [30] (Table 2).

### 3.5. ROS Generation and Lipid-Peroxidation-Related Analyses

Malondialdehyde (MDA) levels in cells were measured with thiobarbituric acid to assess lipid peroxidation levels [31]. Hydrogen peroxide (H_2_O_2_) levels were assessed as reported by Yu et al. [32], while superoxide anion (O_2_^−^) generation rates were determined as reported by Elstner et al. [33]. Relative conductivity was computed as per Ma et al. [34]. Schiff’s reagent, which is capable of detecting lipid-peroxidation-derived aldehydes, was used for histochemical analyses of lipid peroxidation. Briefly, leaves were placed in Schiff’s reagent for 1 h followed by immersion in boiling ethanol for bleaching until a red/purple color consistent with lipid peroxidation was visible. The histochemical localization of H_2_O_2_ and O_2_^−^ in seedling leaves was assessed per the protocol published by Thordal-Christensen et al. [35], with H_2_O_2_ (brown spot) and O_2_^−^ (dark blue) being colored using solutions of 1% 3,3-diaminobenzidine (DAB) and 0.1% nitroblue tetrazolium chloride (NBT), respectively.

### 3.6. Antioxidant Metabolite Analyses

Oxidized glutathione (GSSG) concentrations were measured as reported previously by Nagalakshmi [36]. Total glutathione and GSSG absorptivity were measured at 412 nm. Reduced glutathione (GSH) levels were calculated based on the difference between total glutathione and GSSG levels.

### 3.7. Antioxidant Enzyme Activity Assays

Tomato leaves (0.3 g) were taken and placed in a pre-chilled mortar, and 3 mL of pre-chilled 0.05 mol L^−1^ phosphate buffer (pH 7.8) was added. It was then ground into a homogenate on an ice bath, centrifuged at 12,000× *g* for 20 min at 4 °C, and the supernatant was separated as the enzyme extract and stored at 4 °C.

The activity of superoxidase dismutase (SOD) was assessed as reported previously by El-Shabrawi et al. [37], with one unit of SOD activity corresponding to the amount of enzyme necessary for a 50% reduction in NBT content as detected at 560 nm in a colorimetric assay. The activity of peroxidase (POD) was detected as reported previously by Cakmak et al. [38] using a 2.9 mL reaction solution containing 0.1 M phosphate buffer (pH 7.0), 0.04 mL of 0.1 M H_2_O_2_, 0.04 mL of 1% guaiacol, and 0.02 mL of the enzyme extraction solution. Absorbance was analyzed for 3 min at 470 nm. Catalase (CAT) activity levels were measured as described by Hasanuzzaman et al. [39]. Briefly, 0.1 mL enzyme extract samples were combined with 1.7 mL of 25 mM phosphate buffer (pH 7.0) and 0.2 mL of 100 mM H_2_O_2_. Changes in absorbance (240 nm) were then assessed within 1 min. Ascorbate peroxidase (APX) activity was measured as in a previous study reported by Nakano et al. [40]. Briefly, 0.1 mL enzyme extract samples were combined with 1.7 mL of 25 mM phosphate buffer (pH 7.0), 0.1 mL of 5 mM AsA, and 0.1 mL of 20 mM H_2_O_2_. Absorbances were read at 290 nm after 1 min. The activity of monodehydroascorbate reductase (MDHAR) was measured as described by Hossain et al. [41], combining 0.1 mL of enzyme extract and 1.7 mL of 25 mM phosphate buffer (pH 7.0), 0.05 mL ascorbate oxidase, and 0.01 mL of 4 mM NADH before measuring the absorbance at 340 nm. The activity of dehydroascorbate reductase (DHAR) was measured as reported by Costa et al. [42] by mixing 0.1 mL enzyme extract samples and 1.7 mL of 25 mM phosphate buffer (pH 7.0), 0.1 mL of GSH, and 0.01 mL of 8 mM dehydroascorbate (DHA) prior to measuring absorbance for 1 min at 265 nm. Glutathione reductase (GR) activity was measured using the method reported by Cakmak et al. [38]. Decreases in the absorbance (340 nm) associated with NADPH oxidation were measured for 1 min, and activity was calculated based on an extinction coefficient of 6.2 mmol L^−1^ cm^−1^.

### 3.8. qPCR

TRIzol was used to extract total RNA from tomato leaves, after which a HyperScriptTM III RT SuperMix for qPCR (EnzyArtisan Biotech, Shanghai, China) with gDNA Remover was used to prepare cDNA based on the provided directions. Then, a 2 × S6 Universal SYBR qPCR Mix (EnzyArtisan Biotech, China) was used for qPCR using appropriate primers (Table 3). Three biological replicate samples were analyzed for each condition, and relative gene expression was calculated by the 2^−ΔΔCt^ method.

### 3.9. Statistical Analysis

Data were expressed as means ± standard deviation (SD) and were compared with SPSS v 19.0 using ANOVAs and Duncan’s multiple interval test. *p* < 0.05 was the significance threshold, and Origin 9 was used to construct all figures in this study.

## 4. Discussion

In an earlier study, we demonstrated that the exogenous application of AsA was sufficient to alleviate salt-stress-induced PSII photoinhibition through both increases in endogenous AsA content and enhanced photosynthetic performance [22]. PSII is generally regarded as the system primarily affected by photoinhibition under stress conditions, but some authors have suggested that PSI may also be susceptible [43,44,45]. Here, significant reductions in *F*_v_/*F*_m_ and *P*_m_ were observed in tomato seedlings exposed to salt stress, suggesting inhibition of both PSI and PSII [46,47]. Salt stress reduced the Y(II) and *qP* of tomato seedling leaves, whereas 1–*qP* was increased, suggesting that excitation pressure increases in PSII (1–*qP*) were attributable to a reduced capacity for CO_2_ assimilation and reduced electron transfer efficiency [48]. Increases in Y(NO) that were observed in salt-stress-exposed plants (Figure 2) suggested the disruption of the PSII supercomplex and/or damage to the D1 protein by excess light energy, which impacts its turnover [49,50]. By applying exogenous AsA, it was possible to mitigate the impact of salinity on Y(II), *qP*, and Y(NO). AsA is reportedly a cofactor of violaxanthin de-epoxidase that is involved in dissipating excess excitation energy from the xanthophyll cycle under stress conditions [51]. Higher AsA levels within plants can drive D1 protein accumulation under conditions of cold stress, for example, thereby alleviating PSII photoinhibition [52]. To further probe the role of AsA, we applied lycorine (an inhibitor of L-galactono-γ-lactone dehydrogenase activity, which is a key enzyme in AsA synthesis) on the leaves of salt-stressed tomato seedlings with or without AsA treatment. LYC was used to reduce endogenous AsA content [53]. In this work, we found that Lycorine treatment could reduce the endogenous AsA content in leaves of salt-stressed tomato seedlings with or without AsA application (Figure S1). Meanwhile, a decrease in Y(II) and qP and an increase in 1–*qP* and Y(NO) were observed under NaCl + lycorine and NaCl + lycorine + AsA treatment compared with salt stress and NaCl + AsA treatment, respectively (Figure 1 and Figure 2). Therefore, Applying AsA may therefore be capable of preserving PSII supercomplex stability or enhancing the turnover of the D1 protein while also augmenting photosynthetic electron transport and Calvin-cycle-associated NADPH and ATP demands [54,55]. Higher *NPQ* values and lower 1–*qP* values following treatment with AsA indicated a reduction in PSII reaction center excitation pressure, an increased capacity for heat dissipation, and a reduction in the degree of photoinhibition [56,57]. In addition, a decreased capacity for CO_2_ assimilation can contribute to a proportion of reduced electron carriers being unable to undergo PSI donor-side oxidation, resulting in the over-reduction of P700 or excess NADP accumulation, which in turn enhances ·OH formation and contributes to PSI damage [58]. Here, Y(I) and [Y(ND)] decreased under stress conditions, whereas Y(NA) increased (Figure 2), thus demonstrating that PSI was sensitive to salt stress and its activity was reduced in relation to P700 over-reduction and PSI impairment. Exogenous AsA application was sufficient to reverse the impact of NaCl or NaCl + lycorine treatment on these indices (Figure 2). AsA was thus able to maintain P700 at a higher oxidation state under salt stress conditions while attenuating ·OH-mediated PSI damage.

State transition is a mechanism through which organisms that rely on photosynthesis can control the relative allocation of excitation energy to PSI and PSII and decrease excitation-energy-related stress in PSII reaction centers [59]. Under adverse conditions, maintaining a balanced distribution of excitation energy is a prerequisite for the efficient operation of PSII and PSI and the coordinated completion of the LEF (linear electron flow) [60]. Here, salt stress was found to result in reductions in *p* and *α* values and increases in *D*, *β*, *Ex*, and *β*/*α*-1, indicating an imbalanced distribution of excitation energy between PSI and PSII. Additionally, an increase in PSII excitation energy stress and the portion of light energy allocated to light reactions (*p*) was significantly decreased, whereas the portion of light energy dissipated as energy from non-photochemical reactions (*Ex*) increased. This may contribute to the reductions in photosynthetic efficiency and PSII damage under salt stress conditions (Figure 3). The application of lycorine led to a further imbalance in the allocation of excitation energy between PSI and PSII in tomato seedlings exposed to salt stress, suggesting reduced regulatory ability of the photosystem energy redistribution mechanism (Figure 3). Exogenous AsA application was able to balance the distribution of excitation energy between PSI and PSII, thus increasing PSII reaction center openness (*qP*) under NaCl and NaCl + lycorine treatment conditions, which allowed more energy for photochemical reactions (*p*) and reduced PSII damage resulting from exposure to excess light energy (*Ex*), as evidenced by *F*_v_/*F*_m_ and Y(II) enhancement. AsA can thus maintain an appropriate distribution of absorbed light energy between PSI and PSII under salt stress conditions, improving the light energy utilization efficiency.

Light energy absorbed by plants is used primarily for electron transfer that drives electron-consuming processes such as photorespiration (PCO) and photosynthetic carbon reduction (PCR) [61]. The total electron flux in PSII [*J*e(PSII)] can be separated into both *J*e(PCR) and *J*e(PCO) as well as the alternative electron flux (*J*a). *J*a comprises both *J*a(O_2_-independent) and *J*a(O_2_-dependent), the latter of which corresponds to the Mehler reaction, which is a key source of ROS such that increased *J*a(O_2_-dependent) can trigger ROS production under adverse conditions [62]. Under normal conditions, PCR functions as a key sink for light energy that has been absorbed, whereas the inhibition of PCR under stress conditions can alter photorespiration, the Mehler reaction, and nitrogen-metabolism-related electron flow [11]. Haupt-Herting et al. [63] found that tomato seedlings exposed to water stress exhibited significant reductions in *J*e(PCR) and *J*a(O_2_-dependent) together with an increase in *J*e(PCR). Moreover, Zhou et al. [64] analyzed PSII electron flux and found that cold-induced reductions in *J*e(PCR) were largely compensated by *J*a(O_2_-dependent) rather than by *J*e(PCO). Here, salt stress was found to inhibit *J*e(PCR) and *J*e(PCO) while enhancing *J*a(O_2_-dependent) and *J*a(O_2_-independent) (Figure 4). These results indicate the trapping of large quantities of excess energy that are then consumed via O_2_ partial-pressure-dependent alternative electron flux, resulting in excessive ROS production. Melatonin and brassinolide (BR) reportedly improve cucumber seedling cold tolerance through mechanisms associated with increases in *J*e(PCR) and decreases in *J*a(O_2_-dependent) [61,65]. Zhang et al. [66] utilized transgenic rice overexpressing l-galactono-γ-lactone dehydrogenase (*GLDH*) and demonstrated that higher levels of endogenous AsA were associated with increases in *J*e(PCR) and *J*e(PCR)/*J*e(PSII) together with reductions in *J*a/*J*e(PSII), implying a role for AsA in photosynthetic electron flow allocation in rice. Here, we found AsA to maintain high *J*e(PCR*)*, *J*e(PCO)/*J*e(PSII), and *J*e(PCR)/*J*e(PSII) levels while significantly suppressing increases in *J*a/*J*e(PSII) and *J*a(O_2_-dependent) in tomato seedlings exposed to salt stress (Figure 5). The application of AsA further reversed the impact of lycorine treatment by inhibiting electron flow allocation to carbon assimilation and inducing Mehler reaction allocation under salt stress conditions. AsA is thus capable of maintaining the ability of PCO and PCR to consume excessive photosynthesis-related electrons, thus reducing O_2_ partial-pressure-dependent alternative electron flux. Excessive light energy can, under stress conditions, contribute to ROS production within chloroplasts that can damage both PSII and PSI. CEF-PSI can effectively protect against photodamage, and many prior reports have demonstrated that CEF can shield PSI from stressors including cold [67], heat [68], drought [69], and other adverse conditions [70]. By oxidizing PSI acceptor-side components through the recycling of electrons from PSI to the plastoquinone (PQ) pool and Cyt b6/f, CEF can mitigate acceptor-side reduction [71]. Here, the exogenous AsA stimulated Y(CEF) in tomato seedlings subjected to salt stress with or without lycorine treatment while improving the Y(CEF)/Y(II) ratio and *J*e(CEF-PSI) (Figure 6). Application of AsA was thus able to stimulate increased CEF activity, transferring PSI electrons to PQ and growing the PQ pool, thus facilitating PQ pool oxidization in these tomato leaves that had been subjected to salt stress. Applying AsA can thus facilitate absorbed light energy distribution to PSI reaction centers, reducing PSI donor-side electron accumulation [72]. In summary, exogenous AsA activated CEF and thereby alleviated PSI photoinhibition under salt stress conditions.

Dysregulated ROS metabolism under stress conditions can cause damage to aerobic organisms and can contribute to photoinhibition resulting from damage to the photosynthetic apparatus. Elevated *J*a(O_2_-dependent) under salt stress or other stress conditions implies enhanced Mehler reaction activity and ROS generation [73]. Here, salt stress increased *J*a(O_2_-dependent) and caused concomitant increases in the accumulation of MDA and ROS (O_2_^−^ and H_2_O_2_) as well as reduced antioxidant enzyme activity levels and increased electrolyte permeability (Figure 7 and Figure 9), indicating that the ROS production capacity was increased and ROS scavenging activity was impaired under salt stress, thereby contributing to oxidative damage. Plants have evolved a range of tightly regulated enzymatic and non-enzymatic mechanisms responsible for the scavenging of ROS, with the CAT, POD, SOD, and AsA–GSH pathways being the most prominent in this context. Both endogenous GSH levels and the GSH/GSSG ratio can offer important insight into redox homeostasis in plants [74,75,76,77]. The ability to maintain such redox homeostasis under stress conditions is critical, as it can shield against damage to the photosystems [78]. Endogenous AsA has been shown to serve as a key non-enzymatic component of these antioxidant defense pathways, as it serves as an electron donor for APX-mediated H_2_O_2_ scavenging in the AsA–GSH cycle when protecting plants against the potential oxidative stress induced by many abiotic stressors [79,80,81]. Exogenous AsA application can similarly improve stress tolerance and promote growth through the detoxification of ROS induced in response to various stressors [82,83,84]. Here, the spraying of AsA on tomato seedlings under salt stress conditions contributed to increases in photosynthetic electron flow, the inhibition of Mehler reaction activity, and reductions in ROS accumulation. Moreover, AsA was associated with improvements in the expression and activity levels of POD, CAT, SOD, APX, GR, DHAR, and MDAR, whereas in salt-stressed plants treated with lycorine, these enzymes were downregulated and suppresses while ROS levels were increased (Figure 7, Figure 9, Figure 10 and Figure 11). Treatment with exogenous AsA similarly promoted increases in the GSH content and GSH/GSSG ratio in the leaves of tomato seedlings exposed to NaCl or NaCl + lycorine treatment (Figure 8). These data further confirmed that exogenous AsA was able to increase photosynthetic electron flow, inhibit ROS production, enhance the detoxification of ROS, and maintain redox homeostasis in salt-stress-exposed plants. This pathway is one of the key mechanisms by which photosystem stability is maintained, and it mitigates photoinhibition in the presence of salt stress.

## 5. Conclusions

**Figure 12:**
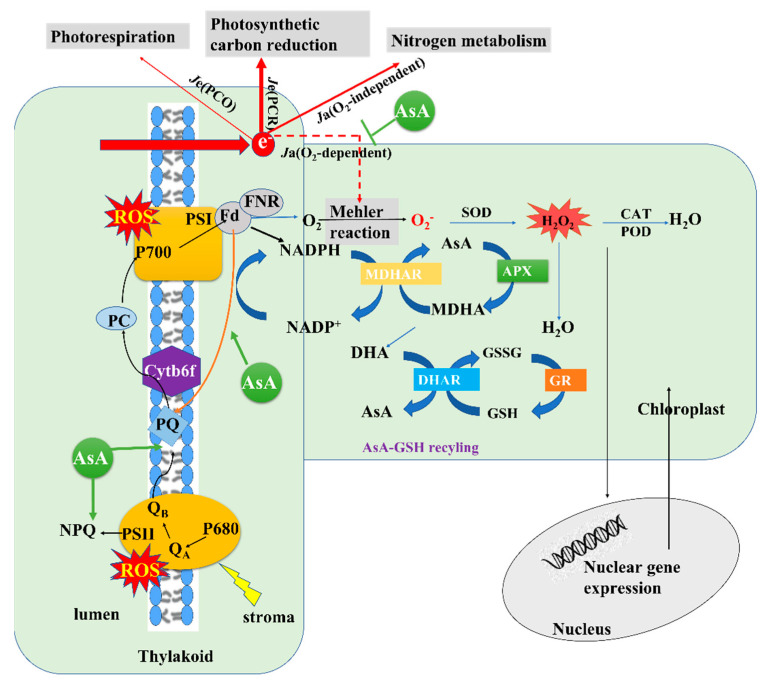
Schematic diagram of the effects of ascorbic acid (AsA) on photosynthetic electron transfer, current distribution, and reactive oxygen species (ROS) scavenging in the leaves of tomato seedlings under salt stress conditions. Note: the solid and dashed lines correspond to promotion and inhibition, respectively, while the relative thickness of arrows denotes an increase or decrease. Electrons generated by photosystem II (PSII) are transferred to photosystem I (PSI) via plastoquinone (PQ), the cytochrome b6/f (Cyt b6/f) complex, and plastocyanin (PC), and they ultimately reduce NADP^+^ to NADPH via Fd (black arrow). In the context of cyclic electron flow (CEF) around PSI, Fd can transfer electrons back to PQ and then back to PSI via Cyt *b6/f* and PC (orange arrow). These electron transfer reactions are coupled with proton pumping into the thylakoid lumen and produce a proton gradient across the thylakoid membrane (ΔpH). AsA promotes an increase in CEF rate under salt stress and NaCl + lycorine treatment conditions, and this increase in CEF rate contributes to the formation of ΔpH, which in turn induces an increase in non-photochemical quenching (NPQ), allocation of photosynthetic electron flux primarily to carbon assimilation and nitrogen metabolism, and a decrease in Mehler reaction electron flow. Increased NPQ induces increased photosynthetic electron flux, which is primarily allocated to carbon assimilation and nitrogen metabolism. This decreases Mehler reaction electron flow and increases the strength of the antioxidant system and the activity of key enzymes in the ascorbate–glutathione (AsA–GSH) cycle. This, in turn, reduces ROS levels, signals to the nucleus, increases gene expression, and provides negative feedback to the chloroplast, which ultimately alleviates oxidative damage to the electron donor and acceptor side of PSII. In addition, the increase in ΔpH maintained the regulation of electron transfer by Cyt *b6/f* and avoided the excessive accumulation of electrons at PSI to reduce the oxidative damage on the acceptor side of PSI.

In summary, these results demonstrate the efficacy of AsA in the mitigation of photosystem inhibition in tomato seedlings under salt stress conditions. The findings showed that AsA could promote the thermal dissipation of PSII excitation energy and mitigate excessive PSI receptor side reduction under salt stress. This was accomplished primarily by balancing the allocation of excitation energy between PSI and PSII, thus activating NPQ mechanisms, photorespiration, and CEF, which maintained the stability of both photosystems and enhanced the quantum yield of PSII and PSI photochemistry. The exogenous application of AsA was also able to promote efficient electron transfer between the two photosystems and protect against photooxidative damage through the enhancement of ROS scavenging and photosynthetic electron flow, thereby effectively alleviating PSI and PSII photoinhibition and improving the photosynthetic performance of tomato seedlings exposed to high levels of salinity (Figure 12).

## Figures and Tables

**Table 1:** Parameters of chlorophyll fluorescence.

Parameter and Formula	Explanation
*F*_v_/*F*_m_	The maximal photochemical efficiency of PSII
*P*_m_	The maximal P700 changes
*NPQ* = (*F*_m_ − *F*_m_′)/*F*_m_′	Non-photochemical quenching coefficient
*qP = (F*_m_′ − *F*_s_*)/(F*_m_′ − *F*_o_′*)*	Photochemical quenching coefficient
1–*qP* = (*F* − *F*_o_′)/(*F*_m_′ − *F*_o_′)	PSII excitation pressure
Y(II) = (*F*_m_′ − *F*_s_)/ *F*_m_′	Effective quantum yield of PSII
Y(NPQ) = (*F*_s_/*F*_m_′) − (*F*_s_/*F*_m_)	The quantum yield of regulated non-photochemical energy dissipation of PSII
Y(NO) = *F*_s_/*F*_m_	The quantum yield of non-regulated energy dissipation of PSII
Y(I) = 1 − Y(ND) − Y(NA)	The effective quantum yield of PSI
Y(ND) = 1 − P700_red_	Fraction of over P700 that is oxidized in a given state
Y(NA) = (*P*_m_ − *P*_m_′)/*P*_m_	Fraction of over P700 that cannot be oxidized in a given state
D = (1 − *F*_v_′/*F*_m_′) × 100%	The fraction of photon energy absorbed in PSII antennae and dissipated via thermal energy in the antenna
*p* = *F*_v_′/*F*_m_′ × *q*_P_ × 100%	The fraction of photon energy absorbed in PSII antennae utilized for photosynthetic electron transport
Ex = *F*_v_′/*F*_m_′ × (1 − *q*_P_)	The estimate of the fraction of excess excitation energy that is neither dissipated in the PSII antennae nor utilized for photochemistry
*Β = 1/(1 + f) and α = f/(1 + f)* *f = (F_m_*′ − *F_s_)/(F_m_*′ − *F_o_*′*)*	β and α represent the photon activity distribution coefficients of PSII and PSI, and *f* represents the opening degree of PSII reaction center
*β/α – 1 = (1 – f)/f*	The relative deviation from full balance (β/α − 1) between PSI and PSII

**Table 2:** Parameters of the linear electron flow (LEF) and cycle electron flux (CEF) transport rate.

Parameter and Formula	Explanation
*J*e(PSI) = Y(II) × PPFD × 0.84 × 0.5	Electron transport rates through PSII. The value 0.5 corresponds to the assumption that excitation is equally distributed between PSI and PSII, while 0.84 corresponds to the general absorptivity of the leaves of C3 plants.
*J*e(PSII) = Y(II) × PPFD × 0.84 × 0.5	Electron transport rates through PSI.
*V*_C_ = (*P*_n_ + *R*_P_)/[1 − *p*O_2_/(2 × *S*r × *C*c)]	The rate of Rubisco carboxylation. *P*_n_ represents net CO_2_ assimilation rate; *R*_P_ represents the rate of day respiration; *p*O_2_ represents the ambient partial pressure of O_2_; *Sr* represents CO_2_/O_2_ relative specificity of RuBisCO; and Cc represents the partial pressure of CO_2_ at the carboxylation site.
*V*_O_ = (*V*_C_ × *p*O_2_)/(*S*r × *C*c)	The rate of Rubisco oxygenation.
*J*e(PCR) = 4 × *V*_C_	The electron flux for the photosynthetic carbon reduction (PCR) cycle.
*J*e(PCO) = 4 × *V*_O_	The electron fluxes associated with photorespiration (PCO) cycle.
*J*a = *J*e(PSII) − *J*e(PCR) − *J*e(PCO)	Alternative PSII electron flux not utilized by the PCR or PCO cycles.
*J*a(O_2_-dependent) = *J*a(21%O_2_) − *J*a(2%O_2_)	Alternative O_2_-dependent electron flux.
*J*a(O_2_-independent) = *J*a(2%O_2_)	Alternative O_2_-independent electron flux.
*J*e(CEF-PSI) = *J*e(PSI) − *J*e(PSII)	Electron transport rates through CEF around PSI.
Y(CEF) = Y(II) − Y(I)	The quantum yield of CEF around PSI.
Y(CEF)/Y(II) = [Y(I) − Y(II)]/Y(II)	The ratio of the quantum yield of CEF around PSI to the effective quantum yield of PSII.

**Table 3:** Sequences of primers used for qRT-PCR.

Gene Name	Primer	Sequence(5′ to 3′)
*Actin* (NM_001323002.1)	FORWARD	TGACTACGAGCAGGAACTTGAAACC
	REVERSE	AACGGAACCTCTCAGCACCAATG
*SOD* (M37151.1)	FORWARD	CGGGTGACCTGGGAAACATAGTG
	REVERSE	ACCACAAGTGCTCGTCCAACAAC
*CAT* (NM_001247898.1)	FORWARD	GCTCCCAGTTAATGCTCCCAAGTG
	REVERSE	CAAGAAGGAATCGGGTACTGCTCAG
*POD* (L13654.1)	FORWARD	GAGAGGTCTGTTCCAATCCGATGC
	REVERSE	TTCGTTGAGTGGTCCATCTACAAGC
*APX* (AY974805.1)	FORWARD	AATTGGCTGGTGTTGTTGCTGTTG
	REVERSE	GGTGGTTCTGGTTTGTCCTCTCTG
*MDHAR* (NM_001247084.2)	FORWARD	GGGTTCTTCTTGAAAGTGGGAGTCC
	REVERSE	GAGCCTCTTCAACCGACGATGC
*DHAR* (NM_001247893.2)	FORWARD	AAGAAGTGGAGTGTGCCTGAAAGC
	REVERSE	AGCCTTGGTTTTCTGGAACGACTC
*GR* (NM_001321393.1)	FORWARD	AGGTTGAATCTGGATGCTGTTGGTG
	REVERSE	AATGCTGGGTATATTGGTGCGTGAG

Note: *Actin* (internal control gene); *SOD* (superoxidase dismutase gene), *CAT* (catalase gene), *POD* (peroxidase gene), *APX* (ascorbate peroxidase gene), *MDHAR* (monodehydroascorbate reductase gene), *DHAR* (dehydroascorbate reductase gene), *GR* (glutathione reductase gene).

## Data Availability

Not available.

## Supplementary Materials

The following supporting information can be downloaded at: https://www.mdpi.com/article/10.3390/plants12061379/s1, Figure S1: Values of reduced ascorbic acid (AsA) content in leaves of salt-stressed tomato seedlings with or without exogenous reduced ascorbic acid (AsA) and lycorine (AsA synthesis inhibitor) spraying. Abbreviations 3d, 6d, and 9d represent the third, sixth, and ninth day after treatment, respectively. Control, no added NaCl and sprayed with distilled water; NaCl, added 100 mmol·L^–1^ NaCl and sprayed with distilled water; NaCl + AsA, added 100 mmol·L^–1^ NaCl and sprayed with 0.5 mmol·L^–1^ AsA; NaCl + lycorine, added 100 mmol·L^–1^ NaCl and sprayed with 0.25 mmol·L^–1^ lycorine; NaCl + lycorine + AsA, added 100 mmol·L^–1^ NaCl and sprayed with 0.25 mmol·L^–1^ lycorine plus 0.5 mmol·L^–1^ AsA. Values are means ± SD (*n* = 3). Values with a different letter within a sampling date are significantly different (*p* < 0.05).
