# Supplementary material for: The Protective Effect of Exogenous Ascorbic Acid on Photosystem Inhibition of Tomato Seedlings Induced by Salt Stress
Source: Plants (Basel). 2023 Mar 20;12(6):1379. doi: 10.3390/plants12061379 (PMC10052531; doi:10.3390/plants12061379)
Supplement: Supplementary file 1 [file plants-12-01379-s001.zip › plants-2253043-supplementary.pdf]

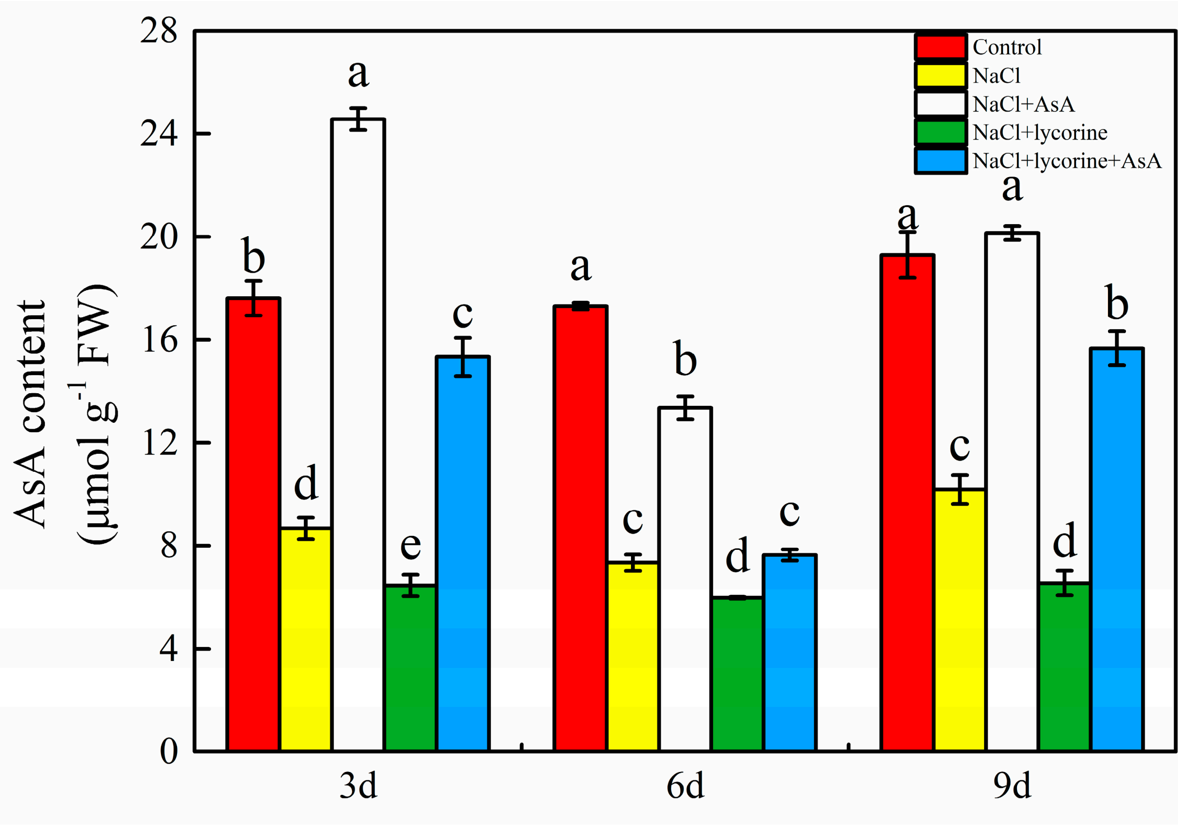

Figure S1. Values of reduced ascorbic acid (AsA) content in leaves of salt-stressed tomato seedlings with or without exogenous reduced ascorbic acid (AsA) and lycorine (AsA synthesis inhibitor) spraying. Abbreviations 3d, 6d, and 9d represent the third, sixth, and ninth day after treatment, respectively. Control, no added NaCl and sprayed with distilled water; NaCl, added 100 $mmol \cdot L^{-1}$ NaCl and sprayed with distilled water; NaCl + AsA, added 100 $mmol \cdot L^{-1}$ NaCl and sprayed with 0.5 $mmol \cdot L^{-1}$ AsA; NaCl + lycorine, added 100 $mmol \cdot L^{-1}$ NaCl and sprayed with 0.25 $mmol \cdot L^{-1}$ lycorine; NaCl + lycorine + AsA, added 100 $mmol \cdot L^{-1}$ NaCl and sprayed with 0.25 $mmol \cdot L^{-1}$ lycorine plus 0.5 $mmol \cdot L^{-1}$ AsA. Values are means ± SD (n = 3). Values with a different letter within a sampling date are significantly different ($p < 0.05$).
